# REST Is Restless in Neuronal and Non-Neuronal Virus Infections: An In Silico Analysis-Based Perspective

**DOI:** 10.3390/v17020234

**Published:** 2025-02-08

**Authors:** Vinod Soman Pillai, Shilpa Ravindran, Gayathri Krishna, Chandran S. Abhinand, Shijulal Nelson-Sathi, Mohanan Valiya Veettil

**Affiliations:** 1 Institute of Advanced Virology (IAV), Bio 360 Life Sciences Park, Thonnakkal P.O., Thiruvananthapuram 695317, India; vinodsoman08@cusat.ac.in (V.S.P.); shilpar@iav.res.in (S.R.); gayathriradhan@iav.res.in (G.K.); abhinandcs@iav.res.in (C.S.A.); 2 Virology Laboratory, Department of Biotechnology, Cochin University of Science and Technology (CUSAT), Cochin 682022, India; 3 Rajiv Gandhi Center for Biotechnology (RGCB), Cheruvikkal Village Office Road, Aakkulam, Thiruvananthapuram 695585, India; shijulalns@rgcb.res.in

**Keywords:** REST/NRSF, herpesvirus, latency, EBV, RE-1 element

## Abstract

Repressor element-1 silencing transcription factor or neuron-restrictive silencer factor (REST/NRSF) is an extensively studied neuronal gene regulator both in neuronal cells and non-neuronal cells. Even though the role of REST in host cellular gene regulation is well established, its role in the establishment of viral infections and its capability to stabilize and destabilize such viral infections are scarcely studied. Co-repressor and DNA modifiers are involved in REST-mediated repressive action of its target genes. The role of REST and co-repressors together or individually in the regulation of viral as well as host genes has been unraveled in a few viruses such as HIV and influenza as well as two of the herpesvirus family members, namely herpes simplex virus type 1 (HSV-1) and Kaposi’s sarcoma-associated herpesvirus (KSHV). Here, we summarize all such virus studies involved with REST to gain a better insight into REST biology in virus infections. We also focus on unraveling the possible RE-1 binding sites in the Epstein–Barr virus (EBV) genome, a well-known human oncogenic herpesvirus that is associated with infectious mononucleosis and neoplasms such as B-cell lymphomas, nasopharyngeal carcinoma, gastric carcinoma, etc. An in silico-based approach was employed towards the prediction of such possible RE-1 binding elements in the EBV genome. This review advances the present knowledge of REST in virus infection which will aid in future efforts towards a better understanding of how REST acts in herpesviruses and other viruses for their infections and pathogenesis.

## 1. Introduction

Recognized in 1995, the multi-zinc finger transcription factor REST/NRSF (Repressor element-1 silencing transcription factor, also known as neuronal restrictive silencer transcription factor) has ever since been described as a transcription factor of importance in neurobiology [1,2,3]. The role of REST ranges widely in neurons from neurogenesis to neural plasticity, differentiation and brain development by means of synaptic plasticity, cellular death evasion and survival by aging, as well as restricting genes involved in oxidative and other exogenous stress [4,5,6,7,8,9,10,11]. REST is known to actively repress various neuronal genes, both coding and non-coding ones that are involved in overall undifferentiated neuronal stem cell functions as well as during terminal neuronal differentiation [1,2]. Although extensively identified to be a neuronal gene-specific repressor in neurons, it is now recognized as a neuronal gene repressor in non-neuronal cells [12]. Numerous studies on REST have unraveled its role in regulating the genes involved in neuronal senescence [10]. Co-immunoprecipitation and in silico analysis have proven that REST is indeed a master transcriptional regulator or hub of neuronal genes, with a regulatory role accounting for approximately 2000 genes and thereby redefining the cellular fate [13,14]. Impaired regulation of REST may result in ischemic responses and seizures as well as other major neurological disorders, evidenced by excessive nuclear localization in Huntington’s disease and its loss in Alzheimer’s [11,15,16,17,18]. REST, being a transcriptional repressor, mediates its role in the neuronal and non-neuronal cellular physiology by binding to the repressor element-1 or neuron-restrictive silencer element (RE-1/NRSE), the REST binding nucleotide sequence found within the promoter or transcriptional regulatory region of the genes targeted by REST.

Although a highly studied transcriptional regulator, REST in virus infection scenarios has not been well explored. REST is implicated in various roles in latency establishment, negative gene regulation of viral genes on infection as well as in aiding protection of the host from infection [19,20]. REST and co-repressors are studied in viruses such as human immunodeficiency virus (HIV), influenza virus, herpes simplex virus type-1 (HSV-1), and Kaposi’s sarcoma-associated herpesvirus (KSHV). The role of REST varies at different stages of their life cycle. REST downregulates viral genes during HSV-1 infection, while sialylated IgG leads to the upregulation of REST which suppresses and protects the host from NFkB mediated inflammation in influenza infection [20,21]. REST degradation via Kaposin is observed in KSHV infection, and the REST-associated co-repressor Co-REST is recruited for silencing HIV infection by Nurr1 [22,23,24]. The role of REST has not yet inspected in another well-known herpes virus, Epstein–Barr virus (EBV), which is known to exhibit both latent and lytic life cycles, associated with several human cancers, and potentially in various autoimmune disorders. In the context of this review, our in silico analysis for prospective REST binding sites (RE-1 elements) in the EBV genome demonstrates that potential non-canonical RE-1 binding sites exist within the exon of the EBNA2 gene. As EBNA2 is a key transactivator of cellular transformations mediated by EBV [25,26,27], this new insight could aid in gaining a better understanding and direction for future studies based on the EBV life cycle. This could also bring more momentum in developing antivirals specifically able to target EBV infection and its associated human diseases including malignancies.

## 2. REST Repression Mechanism and Neurons

### 2.1. REST Protein

**Figure A1:**
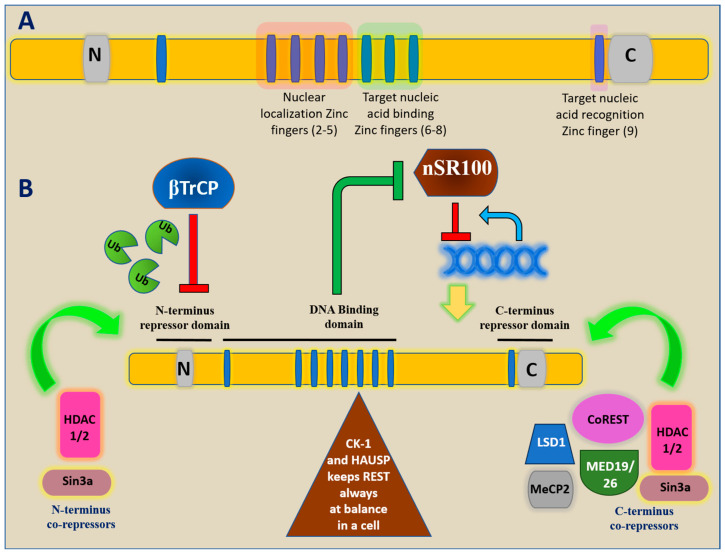
(**A**) The REST protein structure (yellow bar) with a carboxy terminus (C), amino terminus (N), and all nine zinc fingers (blue domains) is denoted with all of its functions. (**B**) REST is maintained at a balanced level within the cells by CK-1 and HAUSP, the disruption of which may lead to infections and manifestations. REST protein is degraded by β-TrCP-mediated E3 ubiquitin ligase. nSR100 regulates the REST gene through alternative splicing.

REST, which belongs to the Kruppel-like zinc finger family of proteins, is a 1069 amino-acid-long 116 kDa protein [1,2,28]. REST has high homology among species and carries nine Cys/His2 zinc finger domains, a lysine rich region, a proline rich region, and has two nuclear localization signals (NLS) in the lysine-rich region and in the zinc finger 5. REST has two repressing domains, both at the carboxy terminus (56 amino acids) and the amino terminus (83 amino acids) (Figure A1A) [29,30,31]. Nuclear localization of REST is accomplished by zinc fingers 2 to 5, while binding is facilitated by zinc fingers 6, 7, and 8. The target nucleic acid segment recognition is made by the zinc finger 9 [29]. REST has four alternative splice variants with different combinations of domains, wherein the variant-lacking carboxy terminus repressor works opposite to the repressive nature of the full-length variant, resulting in the transcriptional activation of bound genes [32]. REST splice variant levels determine the cell’s fate as variants counteract and lead to differential expression of their target genes and subsequently alter cellular activity [33,34,35].

### 2.2. Repressor Element 1 (RE-1) Binding by REST: REST Repressor Domains, Co-Repressors, RE-1 Binding Elements, and Epigenetic Modifications

First described in superior cervical ganglion gene (SCG10) and voltage gated type II sodium channel in 1995, REST regulates the expression of its target genes by binding to the RE-1 element, a 21 base nucleotide sequence [1,2]. The RE-1 element is split into different halves, right and left, and can be separated by up to 3–16 non-conserved nucleotides and a minimal 2 nucleotide separation. Based on the number of non-conserved nucleotides between the two halves, two types of RE-1 elements exist: canonical and non-canonical RE-1 elements. The canonical RE-1 element contains the right and left halves separated by 2 non-conserved nucleotides, whereas the non-canonical RE-1 element contains the bipartite halves separated by nucleotides varying in length from 3 to 16 bases. REST significantly binds to the 21 base canonical RE-1 element, emphasizing that the two halves are only to be separated by 2 nucleotides [36,37]. Although canonical consensus RE-1 elements are the most favorable binding site for REST, non-canonical binding sites also exist with varying orientation of the bipartite halves [38,39]. They may differ as well in the binding sequence makeup from the actual canonical consensus binding RE-1 element. [13,40,41]. REST mediates the repressive activity of each target gene by recruiting various co-repressor molecules otherwise known as REST repressor complexes to the RE-1 element [9,42]. REST can independently mediate its repressive role with either or both the amino and carboxy termini from proximal and distal positions.

REST mediates the transcriptional repression in its target genes by epigenetic modifications by means of recruitment of DNA-modifying proteins such as histone deacetylase and methyl transferase. The carboxy and amino termini do this independently by recruiting co-repressor complexes to two repressor domains located on the amino and carboxy termini. The amino terminus-based repression is accomplished by the recruitment of co-repressor molecules Sin3a and HDACs [6,43,44], whereas the carboxy terminus recruits co-repressors such as the co-repressor for element-1 silencing transcription factor (Co-REST), paired amphipathic helix protein Sin3a, methyl CpG2 binding protein (MeCP2), HDAC1 and 2, C terminal binding protein (CtBP), site-specific demethylase LSD1, and mediator complex subunits MED19 and MED26 (Figure A1B) [45,46,47,48,49,50]. REST-mediated recruitment of HDAC1 to the RE-1 element results in acetyl group removal of the histone, thereby tighter chromatin packing, ensuring inaccessibility to gene transcription systems for their binding sites. Apart from the co-repressors, DNA modifiers such as G9a histone methyltransferase do a long-term similar job by methylating the 9th lysine residue of histone 3. This enables a high affinity binding site for heterochromatin protein 1 (HP1) to the methylated lysine site of histone 3 (H3K9), facilitating HP1 oligomerisation and higher order compaction of the heterochromatin, thereby reducing accessibility to other binding molecules. Repression is also ensured by the repositioning of the nucleosome by REST-mediated recruitment of the chromatin remodeling enzyme BRG1 [51,52].

REST not only mediates the repression of genes, but also manages to activate genes by recruitment of proteins such as TET3, where the TET3 forms a complex with REST and also mediates the recruitment of NSD3, a histone methylase, resulting in the activation of the RE-1 element-carrying gene to be activated [53]. Although repression of the RE-1 element is occurs through the collective action of the co-repressors and DNA modifiers, their specific recruitment to the repression site by REST varies widely based on cellular type and state. Repression of RE-1-carrying genes by REST is highly dependent on REST levels within the cell, as evidenced by the lower levels of REST in mature neurons compared to non-neuronal cells or neuronal stem cells. Higher REST levels in non-neuronal cells result in tighter repression and regulation due to inaccessibility to the target genes by other transcription activators, while in mature neurons the target genes are found open and available to be transcribed more often due to lower REST levels within the cells [54,55]. Higher levels of REST along with its co-repressors and DNA-modifying interacting molecules have thus been implicated in neuronal development, neurogenesis, neuronal diseases, and neuron survival. REST also plays a key role in regulating neuronal gene expression in non-neuronal cells [56,57].

### 2.3. REST Regulation

Intracellular levels of REST have been documented to be protective in contrast to its role in augmenting cellular death. This has been evidenced in the cortical and hippocampal regions of healthy brains undergoing aging, where REST plays a pivotal role in maintaining cognitive function and protecting the neuronal cells from oxidative stress by repressing the particular genes involved [58,59]. REST has also been mentioned as an augmenter of disease progression in the case of stroke and epilepsy, suggesting that REST cellular levels are critical [60,61,62]. REST levels are kept in check by HAUSP-dependent deubiquitination and casein kinase 1 (CK-1)-involved SCF (Skp1-Cul1-F box protein)/β-TrCP (beta transducin repeat containing protein)-dependent ubiquitin-based proteasomal degradation (Figure A1B) [63,64,65,66,67]. The E3 ubiquitin ligase β-TrCP binds to REST and similar other target proteins such as β-catenin and nuclear factor-kB (NF-kB), further initiating ubiquitination and degradation [68,69]. The carboxy terminus of REST naturally carries two neighboring distinctly natured non-canonical degron motifs, identified to be regulated by an upstream kinase that determines REST destiny in a cell [70]. It is now known that CK-1 mediates the phosphorylation of serine residues within the degron motif, which leads to recognition of the phosphoserine moieties by E3 ubiquitin ligase. ERK1/2-mediated REST protein regulation by phosphorylation of proline rich regions upstream to the degron motifs can be detrimental in differentiating neurons. Studies have evidenced peptidylprolyl cis-trans isomerase Pin1 recruiting β-TrCP to the phosphorylated Ser 861/864 sites within REST and further leading to degradation of REST [18,71]. REST levels are depleted in differentiating neurons by higher levels of β-TrCP and CK1. In contrast, higher REST levels counteract susceptible neurons in the hippocampus due to lowered β-TrCP and CK1 levels [11,17].

A proteasomal independent regulation of REST also occurs in neurons. The neural-specific Ser/Arg repeat related protein nSR100/SRRM4, a 100 kDa protein, is capable of negatively regulating the expression of REST [35,72]. nSR100 directly binds to the REST regulatory region, thereby leading to neuron specific splicing and later increasing the expression of low repressive REST and uplifting the repression of neuronal genes. Conversely, in non-neuronal cells, REST in turn represses nSR100, thereby ensuring the repression of neuronal genes (Figure A1B) [35,73].

REST can also be downregulated by cytoplasmic translocation, as evidenced in autophagy resulting from serum starvation in neuron associated cells. This could be attributed to ubiquitin-mediated degradation in dysregulated scenarios such as Alzheimer’s. Additionally, REST is found to be co-localized with amyloid beta peptide (Aβ) in autophagosomes [59,74]. Also, in Huntington’s disease, REST is believed to be sequestered to the cytoplasm due to the complex formation between cytoplasm residing huntingtin and huntingtin-associated protein 1 (HAP1) further interacting with REST-interacting LIM domain protein (RILP), while this is reversed in mutant huntingtin mouse models leading to RLIP mediated enhanced translocation of REST to nucleus and repressive action [75,76,77]. Collectively, REST levels in cells have to be regulated and kept in balance to maintain cellular homeostasis. (Figure A1B) [78].

### 2.4. REST Biology in Neurons

REST is implicated in the self-renewal of pluripotent stem cells and neurogenesis. Apart from neuronal differentiation, REST also plays a crucial role in the synaptic activity of mature neurons. Several synaptic and extra-synaptic proteins are handled by REST, such as the NMDA receptor (NMDAR) and KCl cotransporter KCC2 which are modulated by REST. NMDARs are responsible for various functions within the brain and neurons which enable neural circuit formation, synaptogenesis and memory formation, as well as learning. REST is capable of switching to the NMDAR GluN2 subunit, thereby regulating even synaptic plasticity, maturation, and memory formation [10]. Moreover, the REST-mediated switch of NMDAR subunits GluN2A and GluN2B results in mature (GluN2A) and immature (GluN2B) phenotypes in the synapses found within the brain’s hippocampus. GluN2A leads to less AMPA-type glutamate receptors (AMPARs) being incorporated into synapses and thereby improving the hippocampus-dependent learning process. This is also significant in the case of the chloride transporter KCC2 and NKCC1, where NKCC1 is highly expressed in immature neurons while KCC2 is repressed by REST, which leads to a balanced inward and outward flow of chloride ions to maintain its higher intracellular concentration [79].

REST regulation is critical in GABA-associated synaptic neurotransmission. During neuronal maturation, the lowered effect of REST on the KCC2 gene consequently reduces the NKCC1-mediated chloride movement, thus hyperpolarizing the neurotransmitter GABA. Similarly, during neurodegenerative disorders and neuronal insults, deregulated expression of REST results in the repression of various post-synaptic and presynaptic proteins are repressed, such as synapsin I, synaptophysin, synaptogamins II, IV, VI, and VII, synaptobrevin II, as well as SNAP25, a SNARE protein [80,81]. The scenario varies in the aging neurons, as lower REST expression protects such neurons and, in many cases, loss of REST expression leads to Alzheimer’s disease [82]. REST was evidenced to protect mice brains from neurotoxin challenge compared to REST ablated controls [83,84]. Additionally, REST is implicated to have a role in neurological disorders such as Huntington’s disease, Parkinsons, and also X-linked neurological retardation diseases such as SMCX [4]. Altogether, a regulated REST expression is critical in neuronal survival, neuronal plasticity, synaptic transmission, and learning, as well as protection from death and aging.

### 2.5. REST in Virus-Infected Neuronal Cells

HSV-1 is a neurotropic virus that establishes latency in peripheral neurons. Studies on HSV-1 reveal the presence of an RE-1 element between the promoters for immediate early genes ICP22 and ICP4, which suggests the role of REST in the establishment and maintenance of HSV-1 latency. REST was found to inhibit the HSV-1 ICP4 (infectious cell protein-4) promoter and this inhibition was reversed by the histone deacetylase (HDAC) inhibitor, trichostatin A (TSA), in HEK 293 cells, which results in the recruitment of Co-REST to the ICP4 promoter. This leads to reduced acetylation of histone H4 in the presence of REST [20].

It has also been reported that a short sequence of HSV-1 ICP0, an immediate early protein, is similar to a sequence in the amino terminus of Co-REST. ICP0 binds the REST-Co-REST-HDAC1/2 repressor complex which results in the dissociation of HDAC1/2. Co-REST and HDAC1/2 are phosphorylated by HSV-1 protein kinases and are translocated to the cytoplasm. The dissociation of HDAC1 from the repressor complex by ICP0 indicates that ICP0 has the ability to block the Co-REST-mediated silencing of viral genes [19,85]. Since these experiments were performed using both neuronal and non-neuronal cells, these studies have been greatly helpful in understanding the interesting link between REST and HSV-1-latency establishment.

In a study of REST [recombinant (R) 111] involving a dominant-negative REST (dnREST) lacking the amino and carboxy termini repressor domains (R112) and an insertion control consisting of tandem repeats of stop codons (R113), the recombinant virus R112 carrying the dnREST replicated better and was more virulent than the wild-type parent or the other recombinant viruses when administered by the corneal or intraperitoneal routes. Additionally, the corneal route inoculation by the R112 recombinant virus resulted in higher DNA copy numbers and higher levels of virus, which indicate that the REST/Co-REST/HDAC/LSD1 repressor complex plays a major role in HSV-1 silencing [86]. Furthermore, the expression of dnREST silences the promoters of genes involved in the replication of HSV-1 and also prevents its reactivation in neurons [87]. These studies show the mechanism that regulates the activity of REST in HSV-1 infection, thus highlighting the significance of the REST repressor complex in HSV-1 latency establishment.

The Co-REST transcription repressor complex is involved in silencing human immunodeficiency virus (HIV) in microglial cells. This complex interacts with the epigenetic silencing machinery components such as histone deacetylases 1/2 (HDAC1/2), euchromatic histone lysine N-methyltransferase 2 (G9a; EHMT2), lysine (K)-specific demethylase 1A (KDM1A), and the enhancer of zeste 2 polycomb repressive complex 2 subunits (EZH2) [19,88]. The nerve growth factor IB-like receptor (Nurr1) recruits the Co-REST transcription repressor complexes and regulates inflammatory cytokine synthesis, which includes TNF-α and IL-1β, following the stimulation with LPS [89,90]. Moreover, the protein level analysis of Co-REST knockdown (KD) revealed that Co-REST KD has the ability to prevent HIV silencing, which indicates a role of Co-REST repressing complexes in HIV infections [24].

## 3. REST in Non-Neuronal Cells

REST plays a crucial role in the development process and disease of various organs such as the heart, pancreas, skin, and eye. Apart from these tissues, the role of REST is extensively studied in various cancers where it exhibits a dual function, both as a tumor suppressor and tumor promoter. A decreased expression of REST is observed in non-neural tumors and acts as a tumor-suppressor, whereas an increased level of REST expression is observed in neural tumors and acts as a tumor promoter [67].

### 3.1. Tumor-Suppressive Role of REST

Loss of function of REST was observed in 20% of breast cancer, which is associated with aggressive phenotype and poor prognosis [91]. REST knockdown in breast cancer cells leads to the significant upregulation of KIAA1199/CEMIP (cell migration-inducing, hyaluronan-binding protein) and MMP24 (matrix metallopeptidase 24), genes which are involved in cell invasion and metastasis [91].

In addition, a genetic screening of triple-negative breast cancer subtype (TNBC) demonstrated the presence of STP axis components (SCYL1, TEX14, and PLK1) which degraded the REST by phosphorylating a conserved REST phospho-degron and associated the REST interaction with ubiquitin-ligase β-TrCP. Moreover, the inhibition of STP axis components resulted in high expression of REST proteins and impairment of TNBC transformation, which indicated the tumor-suppressive role of REST in breast cancer cell lines [92].

An array-comparative genomic hybridization (CGH) analysis in colorectal cancer revealed that REST is a frequent target of deletion in colorectal cancer (CRC). This analysis also identified a frameshift mutation in the REST gene which encoded a dominantly acting truncation and caused transformation of epithelial cells. Increased PI3K signaling was also observed in cells which lack REST expression and was attributed to their transformed phenotype which clearly depicted the tumor-suppressive role of REST in CRC [93].

In small-cell lung cancer (SCLC) cell lines, a low-level expression of REST was observed which resulted in the expression of neuronal markers like the L1-cell adhesion molecule (L1-CAM) and neural cell adhesion molecule (NCAM). Apart from its regulated genes, REST itself could promote tumor suppression by methylation. In SCLC’s, the potential upstream regulation of REST occurs by methylation and cyclic adenosine monophosphate response element binding protein (CREB). Downstream investigation revealed that REST directly regulates AKT2, where loss of REST can lead to an epidermal growth-factor-mediated deregulation of AKT-Serine 473 phosphorylation, an important process required for cellular proliferation and survival [94]. Another study showed that KDM1A, or lysine-specific histone demethylase 1 (LSD1), a demethylase of the mono- and di-methylated histone H3 lysine 4 (H3K4me1/2) was significantly upregulated while REST was inactivated in human SCLC specimens. KDM1A functions as an upstream transcriptional regulator that demethylates H3K4me2 at the REST promoter region. Thus, the tumor suppressor REST is transcriptionally inactivated by KDM1A to promote SCLC progression. Knock-out of KDM1A (KDM1A-KO) or treatment with KDM1A demethylase inhibitors resulted in the up-regulation of REST along with the suppression of cancer cell growth [95]. All these studies clearly indicate the role of REST in tumor suppression and growth inhibition in cancer.

### 3.2. Oncogenic Role of REST

As discussed previously, REST also acts as an oncogene and promotes cell proliferation in neural tumors. REST expression was found to be significantly high in patients with glioma, a common type of tumors of the nervous system [96,97]. The CRISPR/Cas9 knockout of the REST gene revealed the role of REST in the proliferation of glioblastoma [98]. Moreover, silencing of REST by siRNAs resulted in the reduction in cell proliferation and migration capacities in U-87 and U-251 glioblastoma cells [99]. Yucebas, M. et al. demonstrated that the expressions of REST and RCOR1 genes downregulated SYN1, a transcription factor which represses the expression of neuronal differentiation-related genes. It helps to maintain a cancer stem-like phenotype which contributed to the development of gliomas [100]. Thus, REST could be utilized as a potential therapeutic target for the treatment of glioma patients.

Medulloblastoma is the most malignant brain tumor in the pediatric population. Abnormal expression of REST regulates neurogenesis and is observed in the case of medulloblastoma [101,102]. Both REST and Myc oncogenes are found to be elevated in medulloblastoma by blocking neuronal differentiation and maintaining the “stemness” of these cells [103]. The upregulation of REST in medulloblastoma is attributed to the increased secretion of proangiogenic transcription factor E26 oncogene homolog 1, and its target gene encoding the vascular endothelial growth factor receptor-1 (VEGFR-1). VEGFR-1 allows vascular growth and also displays molecular and functional features of endothelial cells, suggesting that REST may alter cell fate decisions in medulloblastomas by modulating the expression of transcription factors that control angiogenesis [103]. In addition, REST mediates cancer progression in medulloblastoma by chromatin remodeling, epigenetic repression of the genes that encode PTCH1 and PTEN, thereby enhancing proliferative and migratory signaling by sonic hedgehog (SHH) and AKT activation [104]. Another study revealed that pioglitazone, a peroxisome proliferator-activated receptor-γ (PPARγ) agonist and an antihyperglycemic drug against type 2 diabetes as well as an antineoplastic drug, inhibit proliferation and induce apoptosis of glioma cells by down-regulating REST mRNA [105]. Moreover, elevated levels of lysine-specific demethylase 1 (LSD1) and REST are observed in medulloblastoma, which promotes medulloblastoma cell migration. LSD1 inhibition of REST blocks the REST-dependent cell migration of medulloblastoma cells [106]. Collectively, these findings shed light on the crucial role of REST in medulloblastoma.

Neuroblastoma is the most common and deadly solid tumor affecting the pediatric population. The expression level of REST is found to be significantly high in neuroblastoma as well [107,108]. The enhanced REST activity in neuroblastoma is found to be associated with higher clinical stages and the loss of heterozygosity on chromosome 11q23, a feature of high-risk neuroblastomas [107]. REST expression undoubtedly plays a crucial role in cancer, and is associated with tumor progression-related processes, including cell migration, invasion, and metastasis. More studies are warranted to understand the role and underlying mechanism of REST by molecular and cellular approaches for utilizing it as a prognostic marker and a potent therapeutic target against cancer.

### 3.3. REST in Virus-Infected Non-Neuronal Cells

As REST is a transcriptional regulator, it is predominantly found to be localized in the nucleus of uninfected cells. On the other hand, in Kaposi’s sarcoma-associated herpesvirus (KSHV), REST protein was localized in the cytoplasm of KSHV-infected endothelial cells and B cells by KSHV latent protein kaposin A (K12). Kaposin A mediates the phosphorylation of REST, leading to interaction with the E3 ubiquitin ligase β-TrCP and degradation of REST by the ubiquitin proteasome system. In addition, co-localization of kaposin A and REST was detected in tissue samples from both Kaposi’s sarcoma (KS) and B-cell proliferative primary effusion lymphoma (PEL). These results are indicative of the importance of retention of REST in the cytoplasm of KSHV-infected cells [22]. KSHV-infected endothelial cells and KSHV-infected KS tissue cells are found to express several neuronal and neuroendocrine genes which are regulated by the expression of kaposin A and decreased levels of REST protein [21]. Cumulatively, these results indicate that the downregulation of REST protein is related to KSHV-associated malignancies.

In mouse models expressing human FcγRs (hFcγR mice), the enrichment of sialylated human IgG (hIgG) provided protection against H1 and H3 strains of influenza virus. Additionally, it was observed that this enrichment induced REST expression in the alveolar macrophages (Amϕ). Sialylated IgG-mediated protection was induced by REST in vivo, resulting in the repression of nuclear factor κB (NF-κB)-driven responses against influenza disease. Furthermore, REST induction was confirmed during therapeutic administration of recombinant sialylated Fc molecules in influenza virus-infected patients, offering protection against severe influenza disease. This highlights the protective role of REST in combating influenza virus infections [21,109].

## 4. Identification of REST Binding RE-1 Sites in Viral Genome: An In Silico Insight

REST represses by binding to the 21-base consensus canonical RE-1 repressor elements, while it is also known to bind non-canonical RE-1 elements with both full and partial halves. REST also binds to the sequences, having both halves separated as far as 3–16 nucleotides. It is also observed that the non-canonical consensus RE-1 binding elements may have nucleotide differences when matched with the canonical binding sites. Thus, it is very unlikely to not expect either canonical or non-canonical RE-1 elements while analyzing genomes or sequences for RE-1 repressor elements.

Every virus mediates its lifecycle by utilizing and regulating the host cellular molecules and mechanisms. EBV, the double-stranded DNA virus belonging to the gamma herpesviridae family identified as the first human cancer virus, is known to be associated with cancers like gastric cancer, nasopharyngeal cancers, and lymphomas [110,111,112,113]. EBV infects two types of cells, epithelial cells and B cells, while exhibiting two life cycles of lytic and latent phases [114]. Various host cellular molecules have been identified to interact with EBV during its lifecycle, while exactly how EBV maintains its latent and lytic life cycle is still debated. EBV is hypothesized to harness the host cellular molecules capable of regulating or repressing its genes, thereby mediating different latent phases (0, 1, 2, 3) in infected cells depending on the host cellular conditions [115,116,117]. In silico screening of host cellular molecules capable of conditionally repressing/activating the EBV genome would result in the identification of key host molecules responsible for each latent phase as well as differential EBV oncogene expression. Analysis of known host molecules or possible molecules that may interact with the EBV genome could reveal whether the present understanding of EBV gene regulation could address different latencies or whether any unidentified molecule will form the basis of different EBV latencies. This could also be made possible by screening for host molecules already understood to have a specific role in the isotypic virus life cycle [118].

**Figure A2:**
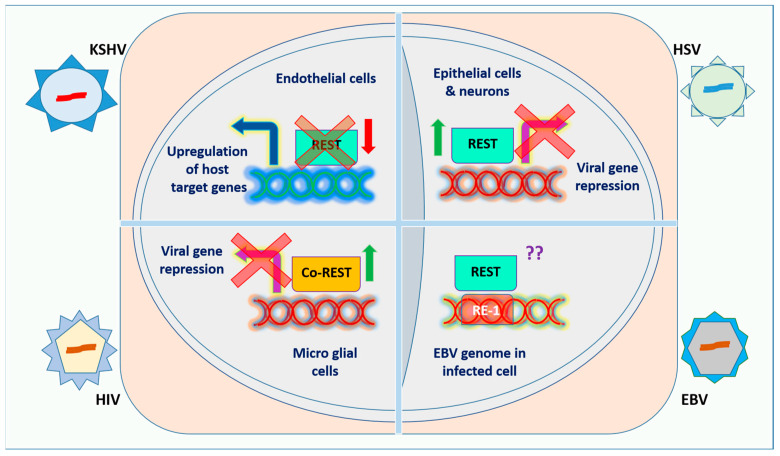
The role of REST and its co-repressors are known in few virus infections. The schematic diagram represents the expression of REST and its regulations in KSHV, HIV, HSV, and RE-1 binding elements in EBV infections. The in silico-predicted possible role of RE-1 element and REST regulation in EBV infection needs to be explored more.

One such candidate molecule of unknown role in the EBV life cycle is REST, which is now understood to have a role in the life cycle or events associated with virus infection, latency maintenance, and, in some cases, even in attaining protection against earlier mentioned viruses such as HSV-1, HIV, influenza, and KSHV (Figure A2). Thus, the screening of the EBV genome for the canonical or non-canonical RE-1 element could reveal the role of REST in the EBV life cycle. A 100% binding efficiency to partial 50% binding could essentially enable REST repression of the RE-1 element and hence a REST binding efficiency analysis in the EBV genome could become critical. Further on, identification of any possible RE-1 element in the EBV genome and specific genes could enable an understanding of how REST could be associated with EBV genes. The efficiency with which REST would bind to the possible RE-1 elements in the EBV genome could better reveal REST’s interaction with its co-repressors and DNA modifiers while also enlightening on the possible mechanism of target EBV gene repression. Essentially, the role of these REST-regulated genes in the EBV life cycle at defined time points will provide an overall understanding of the type of infection that EBV may establish and also help predict its life cycle phases.

We analyzed the role of REST and its interplay with the EBV genome using an in silico methodology. The EBV genome sequence was downloaded from the NCBI database. A custom Python script was used to identify the RE-1 sites on the EBV genome by comparing the canonical (NNCAGCACCNNGGACAGNNNC)/TTCAGCACCACGGACAGCGCC) and non-canonical (NNCAGCACC (NN 3–16 bp) GGACAGNNNC) RE-1 consensus sequences. This was performed on both the forward and reverse strands of the EBV genome separately. To determine the conservation of the RE-1 consensus sequences on the EBV genome, an Ensembl bed file containing the annotated regions was downloaded from the UCSC Genome Browser (https://genome.ucsc.edu/ [accessed on 14 February 2023]). An in-house Python script was used to capture the positions of the identified canonical and non-canonical RE-1 sequences on the EBV genome. The details of the conservation region, including the position and which part of the genome it matched (gene, intron, exon), were fetched.

The analysis enabled the identification of RE-1 elements within the exons of the EBV genome in both forward and reverse directions. While RE-1 element binding sites were found within 17 exon sequences in the forward genome analysis, this was in contrast to only 1 exon sequence in the reverse. Further filtering of the 17 exon sequences possibly carrying the RE-1 element in the forward direction showed that, remarkably, all 16 of the 17 exon sequences corresponded to one particular gene of EBV. This significance was worth noting as the possible binding region being recognized was between bases 37105/8–37128 of the forward genome with only 1 outlier found, although it corresponds to the same exonic region of 37009–37128, but with a longer predicted RE-1 element. Interestingly, another feature of the identified predicted RE-1 binding site was that it had only one or two mismatches from the canonical consensus RE-1 binding site including the outlier. Fascinatingly, the entire above-mentioned 16 predicted non-canonical consensus RE-1 binding region in the forward genome of EBV corresponded to the Epstein–Barr virus nuclear antigen 2 (EBNA2) gene and is tabulated in Table A1.

EBNA2 is a viral transcriptional trans-activator protein capable of regulating the expression of both the EBV latent genes and the host cellular genes. EBNA2 is expressed as a part of EBV viral latency program 3 and is critical for the transformation of EBV-infected B cells in vitro [119,120]. Although EBNA2 is a highly immunogenic protein by itself, EBNA2 transcriptionally activates and readies the primary B cells for transformation by lymphomagenesis and immune evasion [121,122]. EBNA2 activation of miR-24 leads to its target binding of inducible T-cell costimulator ligand (ICOSL), thereby reducing the availability of the ligand to bind the costimulatory CD278 (ICOS) found on primed T cells, T regulatory cells, and follicular T helper cells [123]. Apart from the activation of miR-24, EBNA2 also suppresses the activation of miR34a, leading to the upregulation of programmed death ligand 1 (PD-L1), an inhibitor of immune responses. miR-24 is also known to be involved in c-Myc mediated proliferation and avoiding apoptosis [124].

Identification of the RE-1 element in the EBNA2 gene of the EBV genome holds significance as EBNA2 is implicated in the EBV-mediated transformation of cells. Further studies on the role of the REST repressor complex in the regulation of the EBNA2 gene may also result in identifying mechanisms underlying EBV gene expression, EBV mediated cell growth transformation, as well as the cellular biology of EBV-associated malignancies.

## 5. Conclusions

REST is a master regulator of gene repression in neurons and non-neuronal cells by binding to its repression element RE-1. REST mediates its repression of target genes with co-repressor molecules as wells as DNA modifiers. Loss of REST expression and function either by mutations and alternative splicing or by proteasomal degradation has been implicated in the derepression of REST target genes, which include receptors, peptides, amines, and ion channels. Upregulation of these molecules leads to changes in the regulation of metabolic and signaling pathways associated with different aspects of cancer development, including cell proliferation and tumor growth as well as angiogenesis. In addition to the deregulated REST function in tumor cells, REST is one of the many host factors implicated in the life cycle of HIV, HSV-1, and KSHV.

The role of REST in viral infections is vivid, both in aiding in infection and infection establishment, and in aiding protection against certain viruses. Altering or disrupting the gene expression of crucial regulatory genes within the cells as well as viruses can overturn the mechanism that they are involved in and could also result in altering the cellular fate. There is much scope for unraveling the role of REST in virus infection and such studies still hold importance as REST happens to be a key cellular repressor. Studies in such a direction can still have the potential to create new avenues for drug discoveries to limit viral spread and attain control.

## Appendix A

**Table A1:** The table shows the RE-1 element query conducted for predicting binding sites in the EBV genome both in forward and reverse direction.

Forward EBV Genome Search for RE-1 Element										
Sl No	Sequence Length	Number of N	Number of Mismatch	Query	Hit Sequence	Mismatch Position	Mismatch Pairs	Type	Location in Genome	Gene ID
1	20	1	1	NNCAGCACCNGGACAGNNNC	AGCAGCACCAGCACAGCCAC	[11]	[’G-C’]	exon	36098–37744	“HHV4_EBNA-2”
2	20	1	2	NNCAGCACCNGGACAGNNNC	AGCAGCACCAGCACAGCCAC	[11, 19]	[’G-C’, ’C-C’]	exon	36098–37744	“HHV4_EBNA-2”
3	20	1	2	NNCAGCACCNGGACAGNNNC	AGCAGCACCAGCACAGCCAC	[11, 15]	[’G-C’, ’G-G’]	exon	36098–37744	“HHV4_EBNA-2”
4	20	1	2	NNCAGCACCNGGACAGNNNC	AGCAGCACCAGCACAGCCAC	[11, 14]	[’G-C’, ’A-A’]	exon	36098–37744	“HHV4_EBNA-2”
5	20	1	2	NNCAGCACCNGGACAGNNNC	AGCAGCACCAGCACAGCCAC	[11, 13]	[’G-C’, ’C-C’]	exon	36098–37744	“HHV4_EBNA-2”
6	20	1	2	NNCAGCACCNGGACAGNNNC	AGCAGCACCAGCACAGCCAC	[11, 12]	[’G-C’, ’A-A’]	exon	36098–37744	“HHV4_EBNA-2”
7	20	1	2	NNCAGCACCNGGACAGNNNC	AGCAGCACCAGCACAGCCAC	[10, 11]	[’G-G’, ’G-C’]	exon	36098–37744	“HHV4_EBNA-2”
8	20	1	2	NNCAGCACCNGGACAGNNNC	AGCAGCACCAGCACAGCCAC	[8, 11]	[’C-C’, ’G-C’]	exon	36098–37744	“HHV4_EBNA-2”
9	20	1	2	NNCAGCACCNGGACAGNNNC	AGCAGCACCAGCACAGCCAC	[7, 11]	[’C-C’, ’G-C’]	exon	36098–37744	“HHV4_EBNA-2”
10	20	1	2	NNCAGCACCNGGACAGNNNC	AGCAGCACCAGCACAGCCAC	[6, 11]	[’A-A’, ’G-C’]	exon	36098–37744	“HHV4_EBNA-2”
11	20	1	2	NNCAGCACCNGGACAGNNNC	AGCAGCACCAGCACAGCCAC	[5, 11]	[’C-C’, ’G-C’]	exon	36098–37744	“HHV4_EBNA-2”
12	20	1	2	NNCAGCACCNGGACAGNNNC	AGCAGCACCAGCACAGCCAC	[4, 11]	[’G-G’, ’G-C’]	exon	36098–37744	“HHV4_EBNA-2”
13	20	1	2	NNCAGCACCNGGACAGNNNC	AGCAGCACCAGCACAGCCAC	[3, 11]	[’A-A’, ’G-C’]	exon	36098–37744	“HHV4_EBNA-2”
14	20	1	2	NNCAGCACCNGGACAGNNNC	AGCAGCACCAGCACAGCCAC	[2, 11]	[’C-C’, ’G-C’]	exon	36098–37744	“HHV4_EBNA-2”
15	23	4	2	NNCAGCACCNNNNGGACAGNNNC	ACCAGCAGCACCAGCACAGCCAC	[7, 14]	[’C-G’, ’G-C’]	exon	36098–37744	“HHV4_EBNA-2”
16	24	5	2	NNCAGCACCNNNNNGGACAGNNNC	TACAGCAGCTGGATGTACAGCTAC	[7, 15]	[’C-G’, ’G-T’]	exon	80382–82962	“HHV4_EBNA-3A”
17	29	10	2	NNCAGCACCNNNNNNNNNNGGACAGNNNC	GCCACCACCAGCAGCACCAGCACAGCCAC	[4, 20]	[’G-C’, ’G-C’]	exon	36098–37744	“HHV4_EBNA-2”
Reverse EBV genome search for RE-1 element										
**Sl No**	**Sequence length**	**Number of N**	**Number of mismatch**	**Query**	**Hit sequence**	**mismatch position**	**mismatch pairs**	**Type**	**Location in genome**	**Gene id**
1	31	12	2	NNCAGCACCNNNNNNNNNNNNGGACAGNNNC	GGCAGCGCCCGGGCCACCCGGGGAGAGTTCC	[6, 24]	[’A-G’, ’C-G’]	exon	168749–169056	“HHV4_LMP-1”
